# Measuring Resilience in Women with Endometriosis

**DOI:** 10.3390/jcm10245942

**Published:** 2021-12-17

**Authors:** Daniel María Lubián-López, Davinia Moya-Bejarano, Carmen Aisha Butrón-Hinojo, Pilar Marín-Sánchez, Marta Blasco-Alonso, Jesús Salvador Jiménez-López, Emilia Villegas-Muñoz, Ernesto González-Mesa

**Affiliations:** 1 Department of Obstetrics and Gynecology, University Hospital of Jerez de la Frontera, 11407 Cádiz, Spain; dmlulo@gmail.com; 2 Department of Obstetrics and Gynecology, School of Medicine, University of Cádiz, 11003 Cádiz, Spain; 3 Department of Obstetrics and Gynecology, Regional University Hospital of Málaga, 29001 Málaga, Spain; martablascoalonso@gmail.com (M.B.-A.); evillegasm@sego.es (E.V.-M.); egonzalezmesa@gmail.com (E.G.-M.); 4 Department of Obstetrics and Gynecology, Quironsalud Campo de Gibraltar Hospital, 11379 Cádiz, Spain; aisha.butronhinojo@gmail.com; 5 Department of Obstetrics and Gynecology, University Hospital Virgen de la Arrixaca, 30120 Murcia, Spain; udmog_sms@carm.es

**Keywords:** endometriosis, resilience, psychological disease

## Abstract

Endometriosis is a multifactorial disease with pathophysiological factors not yet well known; it also presents a wide symptomatic range that makes us think about the need for multidisciplinary management. It is a chronic disease in which there is no definitive treatment, and is associated in a large majority of cases with psychological pathology. Connecting comorbidities and multimorbidities on a neurobiological, neuropsychological, and pathophysiological level could significantly contribute to their more successful prevention and treatment. In our study, resilience is analyzed as an adjunctive measure in the management of endometriosis. *Methods*: A multi-centre, cross-sectional study was performed to analyse resilience levels in a sample of Spanish women suffering from endometriosis. CDRIS-25, CDRIS-10, BDI, the STAI, and the SF-36 Health Questionnaire were used for assessments. A representative group of 202 women with endometriosis was recruited by consecutive sampling. Exploratory and confirmatory factor analyses were performed for both resilience scales. *Results:* Mean CDRIS-25 and CDRIS-10 scores were 69.58 (SD 15.1) and 29.37 (SD 7.2), respectively. Women with adenomyosis and without signs of deep endometriosis showed the lowest scores. The best predictive model included women’s age, years of endometriosis evolution, number of pregnancies, and history of fertility problems as the best predictive factors. *Conclusions*: Women build resilience as the number of years of evolution of the disease increases. Symptoms such as dyspareunia and continued abdominal pain were more prevalent among less resilient women.

## 1. Introduction

Endometriosis is a condition defined as a benign and proliferative disorder characterized by the ectopic presence and growth of functional endometrial tissue, glands, and stroma outside the uterine cavity [1]. It is a chronic inflammatory disease and one of the most common gynaecological issues. According to epidemiological data, the incidence of endometriosis in the general female population varies between 4% and 15%, depending on the source [2,3]. As endometriosis may have a subclinical course, the real prevalence may be underestimated. Nevertheless, it has been reported in up to 50% of women suffering from infertility [4]. The clinical features of endometriosis are variable and unpredictable in both presentation and course. Affected women usually present with pain and infertility during their reproductive years [5,6].

Pain is the common thread in all clinical endometriotic situations; it can manifest in different ways, depending on the localization and timing of lesions [7].

The impact of pain is dynamic; it is experienced in a subjective and multifaceted way, the comprehension of which necessitates a good description of its features in each individual patient. ‘Perceived’ pain seems independent of disease stage: women with only mild endometriosis who suffer from disabling painful symptoms can be observed, and vice versa.

Regardless of the pathophysiological pathways of pain, affected patients present a marked psychosocial vulnerability [8]. Especially, those with pelvic pain frequently present psychiatric disorders such as depression and anxiety [9,10,11,12]. There is, in particular, a tendency to develop affective or anxiety disorders, as well as panic-agoraphobic and substance use disorders. Endometriosis with pelvic pain, infertility, and psychiatric vulnerability usually leads to disability and a markedly lower quality of life (QoL) for women of reproductive age [13,14,15,16]. Thus, the burden of endometriosis is not limited to the symptoms and dysfunctions of the disease; it extends to the social, work, and emotional spheres, leading to severe impairment of global functioning.

Health-related quality of life (HRQoL) [11] is influenced by chronic diseases [12] and published clinical studies have demonstrated that women affected by endometriosis have worse HRQoL than those in the healthy population [17,18,19,20,21]. Some of them revealed a close relationship between specific temperamental traits, the expression of several psychiatric symptoms, chronicity of pain, the risk of substance use, and a lower probability of a positive outcome. Biopsychosocial models have been proposed to explain the strong association between chronic pain, altered HRQoL, and psychological factors such as catastrophizing thoughts, with pain and psychological distress inducing negative effects on cognitive functioning and well-being [19].

As a positive emotional resource, resilience could be useful for improving HRQoL, especially in vulnerable groups, and the identification of its predictive factors would be beneficial for any health system. Resilience is an important element in the experience of pain and disease, as it allows adaptation to suffering, and increases social and psychological wellbeing. Patients’ resilience has been investigated in cancer and non-cancer chronic painful conditions, such as fibromyalgia, rheumatoid arthritis, systemic lupus erythematous, and musculoskeletal pain, but to our knowledge, no information exists on resilience in women with endometriosis [22].

The most common definition of resilience is the ability to cope with significant change, adversity, or risk, and thrive in the face of adversity [23]. Resilience is a positive adaptation against adversity, and therefore considers two distinct dimensions, significant adversity and positive adaptation [24,25,26], and it allows patients to overcome and positively adapt to significant stressful events, as in the case of chronic disease [27]. In pain medicine, resilience is ‘the capacity to adapt successfully to disturbances that threaten a patient’s viability, function or development’ [28]. It implies the flexible use of emotional resources for adapting to adversity [29], and three types of models have been described to explain how it modifies the effect of adverse vital events, i.e., compensatory, protective, and challenging [30,31].

Endometriosis symptoms and the impact of related psychological consequences, increased vulnerability, and the possible onset of psychiatric symptoms may influence coping strategies, and weaken resilience, thus triggering a vicious cycle leading to a marked deterioration in QoL.

The assessment of a protective factor such as resilience, which represents a complex interaction that leads to positive developmental outcomes of the disease, is proposed.

This research was designed to learn about resilience in patients affected by endometriosis by analysing the clinical and emotional factors that could be associated, and assessing their relationships with type and severity of pain, and psychological distress. The main objective of the present investigation was to report on the resilience of women suffering from endometriosis. To meet this goal, it was necessary to validate the Spanish version of the CD-Risk scale in women with endometriosis. Our secondary objective was to determine if resilience levels were associated with a certain physical symptom of endometriosis (dysmenorrhea, dyspareunia, dysuria, chronic pelvic pain, dyschezia) or with emotional factors.

## 2. Methods

Between 1 January and 30 June 2021, a multi-centre, cross-sectional study was performed to analyse resilience levels in a sample of Spanish women suffering from endometriosis. The diagnosis of endometriosis had been made in accordance with the guidelines of the European Society of Human Reproduction and Embryology (ESHRE), based on the visual detection of endometriotic lesions during previous surgeries, anatomopathological analysis, and typical ultrasonographic features of endometriosis [30]. The study was performed simultaneously in the endometriosis units of three reference hospitals of Spain (Hospital Universitario Virgen de la Arrixaca de Murcia, Hospital Universitario de Jerez, y Hospital Regional Universitario de Málaga). During their on-site medical visit, women were invited to complete an anonymous questionnaire, which included the Spanish validated versions of the Beck Depression Inventory (BDI); the State-Trait Anxiety Inventory (STAI); The Short Form Health Survey (SF-36); the Female Sexual Function Inventory (FSFI); and two versions of the Connor-Davidson Resilience Scale, the short 10-item form (CD-RISC-10), and the expanded 25-item form (CD-RISC-25). We also included a series of questions regarding socio-economic status (monthly household income, and income decreasing by quartile), physical and mental health status, and obstetric background information. For every female participant, a blinded review of their medical records was also performed, so that all the data regarding the evolution of the endometriosis was collected a second time before the answers to the scales were analysed. The inclusion criteria were an age of 18 years or older, sufficient reading skills to complete self-report instruments, and endometriosis symptoms present at the time of assessment. The acceptance of the data protection laws, and the consent form to participate in the study were included.

### 2.1. Instruments

CDRIS-25, CDRIS-10, BDI, the STAI, and the SF-36 Health Questionnaire were used for assessments. The BDI, a self-administered questionnaire, consists of 21 questions evaluated on a Likert-type scale. Cut-off points were set to enable classification of respondents into four groups: 0–13, minimum depression; 14–19, mild depression; 20–28, moderate depression; 29–40, severe depression; and more than 40, extreme depression. The BDI has been validated in Spanish [31]. The STAI [32] is also a self-administered questionnaire, also validated in Spanish [33], composed of two scales, the scores of which define different levels of anxiety, i.e., low (between 0 and 30), moderate (between 30 and 44), and high (over 45). The SF-36 Health Questionnaire [34] is a generic scale that provides a profile of the state of health, and is applicable to both patients and the general population. It is composed of 36 questions (items) that assess both positive and negative states of health. Scores range from 0 to 100, so the higher the score, the better the health. The scale has been translated and validated in Spanish [35]. The FSFI evaluates 19 items on a Likert-type scale, with each item evaluated from 0 to 5 according to the level of agreement or disagreement. The cut-off score for normal sexual function is 26.5 [36,37]. The results regarding sexual function have been previously reported [38], and in order to avoid redundancies, we will not include them in this article. We refer the readers to the previously published work.

The CD-RISC-10 [39] is a self-administered questionnaire made up of 10 items from the original 25-item scale produced by Connor and Davidson [40]. The factor structure of the original 25-item version was unstable across some demographically equivalent samples [41]; therefore, the 10-item version was developed. However, the expanded version has been widely used in populations with medical problems, such as women experiencing infertility [42]. The short CD-RISC-10 version is a 10-item Likert-type scale with five response options (0 = strongly disagree to 4 = strongly agree; 0 = never to 4 = almost always). The score ranges from 0 to 40 in the short form. The CD-RISC-25 version is a 25-item Likert-type scale with five response options (0 = strongly disagree to 4 = strongly agree), with scores ranging from 0 to 100. In both cases, a higher score indicates a higher level of resilience. Both scales have been translated and validated in Spanish [43,44].

On the other hand, the sociodemographic questionnaire included variables regarding the city of residence, women’s age, academic level, working status, and income. Finally, clinical variables regarding obstetric and medical background, family planning, date of diagnosis of endometriosis, type of treatment, number of surgeries, and stage of the disease (stage OMS/EEC) were also recorded.

### 2.2. Population

A total of 368 women diagnosed with endometriosis went to the reference unit for this pathology, and a control was carried out in the study period described. By consecutive sampling, a representative group of 202 patients was recruited (5% standard error, and 95% confidence level). No difference was found in the number of cases contributed by each referral centre. All participants were asked to sign an informed consent form.

## 3. Statistical Analyses

We performed the validation of CDRIS-10 and CDRIS-25. The frequency distribution of the socioeconomic and clinical characteristics was analysed. Subsequently, a bivariate analysis was performed to identify associations between these variables and the scores on resilience, depression, and anxiety. For bivariate analyses, we used the independent sample *t*-test to compare the mean values in two groups/categories of women when conditions of normality were present, and the Mann–Whitney U test in the rest of the cases; in those with a greater number of groups, we used either a single-factor ANOVA or the non-parametric Kruskal–Wallis test according to the conditions of homoscedasticity, which were evaluated using Levene’s test. To compare qualitative variables, the chi-squared test was used. To analyse the relationship between global resilience scores and other quantitative variables (normally distributed), such as depression or anxiety scores or the age of the participants, the Spearman’s correlation coefficient was used. We used logistic regression models to predict the resilience scores, considering the independent sociodemographic obstetric, clinical, and emotional variables considered. The models were constructed using the intro procedure, including the variables that were shown to be significantly associated in the bivariate analysis.

The internal consistency of the resilience scales was evaluated by calculating Cronbach’s alpha coefficient. The Kaiser–Meyer–Olkin (KMO) test and Bartlett’s test for sphericity were performed to assess the adequacy of an exploratory factor analysis (EFA) of CDRISC-25 and CDRISC-10, and subsequently, confirmatory analyses (CFA) were performed. The EFAs were conducted using the analysis of the principal components of the scale, and the Varimax rotation method was used to identify latent factors that explained the observed variance. Structural equation modelling analyses with correlated factors were tested using the maximum likelihood robust estimator. Four fit indices were selected a priori to assess model fit: the comparative fit index (CFI), Tucker–Lewis index (TLI), standardized root mean square (SRMS), and root mean square error of approximation (RMSEA). Acceptable model fit was defined by a CFI ≥ 0.90, TLI ≥ 0.90, and SRMR or RMSEA values ≤ 0.08 [44,45]. On the basis of these criteria, the best fitting final model was selected. Statistical analyses were performed using SPSS Statistics, v 25 (IBM Corp., Armonk, NY, USA). To predict the influence of the sociodemographic, clinical, and psychological variables studied on resilience scores, we used multiple linear regression. The collinearity between factors was analysed to avoid the inclusion of correlated variables in the model. The model was constructed using a stepwise regression procedure, including the variables that were shown to be significantly associated. Also, structural equation modelling analyses with correlated factors were performed using the maximum likelihood estimator.

This study was conducted according to the guidelines of the Helsinki Declaration, and resolution 196/96 of the National Health Council on Research Involving Human Subjects [46]. Approval was obtained from our hospital ethics committee (N/ref.: CEI 2/2020).

## 4. Results

### 4.1. Sociodemographic and Clinical Features

The mean age of participants at the moment of recruitment was 39.5 years (SD 6.8), 21.8% were younger than 35 years-old, 56.28% were between 35 and 45, and 21.4% were older than 45. The mean age at diagnosis of endometriosis was 31.1 years (SD 8.02). Most of the participants were married (81.2%), and only 7.1% reported are single. The academic level was high in the sample, with 50.5% of women with university studies, 34.2% with secondary studies, and 12.3% with elementary school education. Regarding working activity, most of the women were employed (70%), 10% of the participants were housewives, 16% were unemployed, 1% were already retired, and 7% received state sick pensions. Families’ monthly incomes were under 600 euros in 17.1% of the cases, between 600 and 1200 euros in 42.5%, between 1200 and 3500 in 37.3%, and higher than 3500 euros in 3.1% of the participants. Thirty-six per cent of the participants had been on sick leave due to endometriosis at least once after diagnosis, and 11% three or more times. More than 25.8% of the participant women were smokers, and comorbidities were found in more than 42% of the sample, with depression being the most frequent (40.4%), followed by asthma (12.7%), hypothyroidism (8.6%), interstitial cystitis (5.0%), irritable bowel (2.2%), fibromyalgia (1.3%), atopy (1.3%), Vulvodynia (0.9%), ulcerative colitis/Crohn’s (0.9%), and coeliac disease (0.9%). Main variables regarding women´s reproductive history are shown in Table 1. Clinical variables related with endometriosis are shown in Table 2.

### 4.2. Validation of the Resilience Scales

Validation analysis has been included as supplementary material.

We found a Cronbach’s alpha of 0.89 and 0.91 for the CDRISC-25 and CDRISC-10, respectively. The Kaiser–Meyer–Olkin and Bartlett’s sphericity tests were favourable for EFA. All data regarding EFA are presented as supplementary material. For the 10-item scale, only one factor with an eigenvalue > 1 was found. This factor explained 58.0% of the variance. The CDRISC-25 showed a seven-factor structure that explained 65.3% of the total variance. However, as two of these components had only two items each, the factors were restricted and reviewed. A five-factor model was identified that explained 56.4% of the variance. Factor 1 (16.2% of the variance) refers to emotional stability, factor 2 (15.3% of the variance) refers to the effects of previous learning experiences, factor 3 (11.4%) refers to self-efficacy feelings, factor 4 (7.3% of the variance) is related to spirituality, and factor 5 (6.03% of the variance) refers to social interactions and the ability to seek help. A confirmatory factor analysis was performed, and the structural equation modelling analyses confirmed the goodness of fit of this five-factors model as shown in Table S1. The model is represented as shown in Figure S1.

### 4.3. Main Resilience Scores

The mean scores for CDRISC-10 and CDRISC-25 were 69.5 (Std Dev 15.1) and 29.3 (Std Dev 7.1), respectively. Scores ranged from 12 to 40 for CDRISC-10, and from 26 to 100 for the expanded CDRISC-25. We found differences in the distribution of the scores on resilience according to the academic level, women’s income, obstetric history, and current depression, as shown in Table 3. We also found significant differences according to the type of endometriosis: women with adenomyosis and without signs of deep endometriosis being those who showed the lowest scores (46.6, F = 3.9, *p* < 0.001). We found a significant positive correlation between resilience scores (CDRISC-25) and the number of years of evolution of the disease (r = 0.148, *p* ≤ 0.042). Women diagnosed with endometriosis who are part of statewide endometriosis organizations also showed lower resilience scores.

We found significantly higher BDS scores and STAI trait scores in less resilient women, and higher SF36 in more resilient women, as shown in Table 4.

Both resilience scales, CDRISC-10 and CDRISC-25, were highly correlated (r = 0.87, *p* < 0.001). We found significant correlations between resilience scores on both scales and BDS scores (r = −0.4, *p* < 0.001), trait anxiety scores (r −0.28, *p* < 0.001), physical health (r = 0.18, *p* < 0.001), and mental health (r = 0.34, *p* < 0.001). Also, both scales showed that resilient women reported significantly less abdominal pain and dyspareunia. Differences in pain scores according to the CDRISC-25 results are shown in Table 5. The best fitting model included women’s age, years of endometriosis evolution, number of pregnancies, and history of fertility problems as the best predictive factors in resilience (Figure S1 and Table 6).

## 5. Discussion

**Figure 1:**
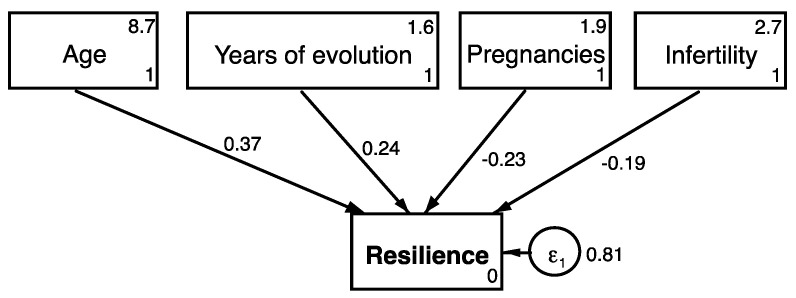
Structural predictive best fitting model.

Our study confirms the usefulness of the Connor-Davidson Resilience Scale (CD-RISC) so that it allows us to identify psychometric factors that can be modified through alternative measures, among others, in order to improve the prognosis of the disease. Within these factors, emotional stability stands out based on the existence of catastrophic thoughts, past experiences based on the time of evolution of the disease, self-efficacy when it comes to developing in relation to fertility and number of children, spiritual status, and social support, as well as the experience of pain (mainly abdominal and sexual pain). As such, our study shows that resilience can be an alternative measure that works as an adjuvant to the treatments we already know (Figure 1).

To this end, the present study investigated the resilience profile in a group of endometriosis patients, and assessed the relationships with the type and intensity of pain, and general distress. We have also presented the validation process of the CDRISC-25 and CDRISC-10 in our sample. To our knowledge, this the first study investigating resilience specifically in patients with endometriosis.

One of the reasons to assess resilience with the expanded and short forms of the CDRISC scale was the great heterogeneity found in previously published studies reporting resilience scores. According to the CDRISC-10 short form, the resilience mean values found in patients with endometriosis were similar to those previously described in the Spanish general population (mean 28.9) [47], or in ART patients after their first or second cycle (mean 28.0 and 28.9, respectively) [48], and slightly higher than initially reported in healthy female first-year university students (26.4) [42]. We also found similar CDRISC-25 scores (mean 69.58) in women with endometriosis than previously described in Spanish general population (mean 70.0) [43,49]. In fact, both scales were very highly correlated.

In the validation of the scales, we found one single factor in the short version of the CDRISC, explaining a higher proportion of the variance (58%) than previously reported [43,46]. Its psychometric properties make it a suitable tool for assessing resilience in women with endometriosis. However, resilience is a multidimensional entity determined by the interplay among hereditary, biological, emotional, intellectual, and external factors [28,31], and a greater number of items, as in CDRISC-25, describe a more complex structure, as found after EFA. As in previous research, a five-factor structure was found in CDRISC-25. In our study, CDRISC-25 provided a more complete vision of the concept, and allowed a more accurate allocation of patients.

Emotional stability (factor 1) has been described as a component of resilience [50]. People with high emotional stability show effective coping strategies, remaining calm and less worried than those with low emotional stability [51]. Emotional stability makes people more future-oriented, and less impacted by present and past experiences [52,53]. As a result, individuals with high emotional stability respond to uncertainty with patience, cope better with unexpected life events, and have good social skills. Moreover, in our study, the presence of catastrophizing thoughts correlated negatively with resilience. In this way, there are several studies which indicated that pain catastrophizing predicted physical and mental health quality-of-life outcomes at discharge, but did not significantly predict clinical pain intensity. Specifically, higher baseline catastrophizing was associated with poorer quality-of-life outcomes [54,55,56].

Being a dynamic process, resilience is built from past experiences (factor 2), and exposures to adversity may either increase or decrease vulnerability to stressors [57,58]. In fact, we found that the number of years of evolution of endometriosis correlated positively and significantly with scores on the CDRISC. Of primary importance for patients with chronic diseases is the potential link between greater difficulties with the regulation of emotions [59,60]. Although we did not find any direct effect of resilience scores on sexual function, this factor correlated significantly and negatively with sexual pain and the emergence of negative cycles of pain that perpetuate the symptom, and impair sexual function, as it is known that most of the sexual effects of chronic disease are negative, and ongoing illness continues to modulate a woman’s sexual self-image, energy, and interest in sexual activity, as well as her ability to respond to sexual stimuli [61,62].

Self-efficacy (factor 3), as one of the components of resilience, is an important tool when facing adversity, and people who believe that they will succeed will be more likely to persevere in their efforts [55]. According to our findings, a higher number of pregnancies were correlated negatively with resilience, most probably due to the physical and psychological overload that a greater number of children produces in women with endometriosis; moreover, infertile women showed low resilience scores. As resilience has been previously reported to correlate significantly and negatively with infertility-related stress [62], and women with low resilience typically show higher levels of infertility-related stress [57], endometriosis patients could benefit from interventions aimed at building resilience.

Spirituality (factor 4) has been previously associated with higher tolerance to psychological and physical stress [63,64].

Social support (factor 5) is important for mental health, and it has been described as an important component of resilience [65]. Our results show that less resilient women attend endometriosis associations or organizations as an attempt to find emotional balance. In pain medicine, resilience is now maintained as an essential element in the experience of pain and in its treatment, as it alleviates suffering, and increases psychosocial well-being and HRQoL; this is included within what is known as integrative medicine, also known as complementary and alternative medicine [66,67,68]. We did not find differences in scores on resilience according to the presence of dysmenorrhea, dysuria, or dyschezia, but we found that more resilient women scored lower on abdominal and sexual pain, which highlights that women would benefit from any intervention aimed at improving resilience. Some authors suggest that a common pathogenetic process could underpin the co-occurrence of chronic pain, mood disorders, and low resilience [7,8,69]. Psychological assessment and follow-up must be considered fundamental tools for the management of endometriosis, and the improvement of the QoL of women affected by the disease [70].

Some weaknesses limit our findings. The sample is relatively small with limited power, and lacks a control group as it is a cross-sectional study; therefore, it was not possible to report causal relationships between resilience and the rest of the variables. However, to our knowledge, there are no previous reports on the effects of resilience in women with endometriosis. We also present the Spanish validation of the short and expanded forms of CDRISC scales in a sample of women with endometriosis.

## 6. Conclusions

In our practice, a multidisciplinary team involving gynaecologists, psychotherapeutists, psychiatrists, specialists in dual disorders, algologists, and sexologists should work unidirectionally to detect, analyse, and, if possible, break the vicious circle by singling out a customized, targeted treatment.

To improve this objective, several scales can be used, such as the CDRISC-10 and CDRISC-25, which have been validated in women with endometriosis. We found that women build resilience as the number of years of evolution of the disease increases. We found that resilience scores in women with endometriosis were similar to those previously described in the general Spanish population, although symptoms like dyspareunia and continued abdominal pain were more prevalent among less resilient women, something that highlights the need for interventions aimed at improving resilience after diagnosis.

These results are the key to considering future prospective studies of larger sample sizes, so that they allow us to know how alternative measures and adjuvants act at the pathophysiological level of the disease, to improve conventional treatments, as well as prognostic factors in endometriosis.

## Figures and Tables

**Table 1:** Reproductive history.

Variable	*N*/(%)
*Pregnancies*	
No	93 (46.5)
1	43 (21.5)
2	49 (24.5)
>3	15 (7.5)
*Miscarriages*	
No	187 (93.5)
1	19 (9.5)
2	12 (6.0)
>2	2 (1.0)
*Ectopic Pregnancies*	
No	212 (96.4)
Yes	8 (3.6)
Deliveries	
Vaginal	67 (72.0)
Caesarean	26 (27.9)
*Desire for Offspring*	
No	72 (61.0)
Yes	46 (39.0)
*Looking for Pregnancy*	
1–2 years	6 (18.2)
2–4 years	8 (24.2)
4–6 years	8 (24.2)
>7 years	11 (33.5)
Use of ART	
Yes	47 (21.4)
No	173 (78.6)
*Number of ART Treatments*	
1	11 (37.9)
2	14 (48.3)
>3	4 (13.8)

**Table 2:** Clinical variables.

Variable	Mean/*N*/(%)
*Years of Evolution*	
Average	7.1 years (SD 5.78)
*Type of Endometriosis*	
Ovarian	77 (38.9)
Deep	23 (11.6)
Adenomyosis	6 (3.0)
Deep and ovarian	53 (26.8)
Deep and adenomyosis	17 (8.6)
Deep, ovarian, and adenomyosis	20 (10.1)
*Diagnosis*	
Clinical	121 (55.0)
Laparoscopic	73 (33.1)
Laparotomic	26 (11.8)
*Pathological Confirmation*	
Yes	126 (57.2)
No	94 (42.7)
*Current Treatment*	
Yes	172 (78.1)
No	48 (21.8)
*Medical Treatments*	
Yes	172 (78.1)
No	48 (21.8)
*First-Line Treatments*	
Contraceptive pills	153 (88.9)
Progestin pills	5 (2.9)
NSAIDs	4 (2.3)
LNG intrauterine device	2 (1.1)
GnRH analogues	2 (1.1)
Other	6 (3.4)
*Rescue Treatments (2nd and 3rd line)*	
Progestin pills	48 (2.7)
Vaginal Progestins	32 (18.6)
LNG intrauterine device	11 (15.2)
GnRh analogues	10 (5.8)
*Complementary Therapies*	
Yes	48 (21.8)
No	172 (78.1)
*Laparoscopic Surgery*	
No	141 (64.1)
Once	60 (27.3)
Twice	13 (5.9)
3 times	4 (1.8)
>3 times	2 (0.9)
*Laparotomic Surgery*	
No	185 (84.1)
Once	27 (12.3)
Twice	6 (2.7)
3 times	2 (0.9)

**Table 3:** Mean values for CDR-25 scores.

Variable	*N* (%)	Mean CDR-25 Scores	
*Academic Level*			
None	6 (3.0)	31.0	F 17.18 *p* < 0.000
Primary	25 (12.4)	68.2	
Secondary	69 (34.2)	70.3	
University	102 (50.5)	71.6	
*Income*			
<600 euros	33 (17.1)	70.3	F 4.70 *p* < 0.003
600–1200 euros	82 (42.5)	66.1	
1200–3600 euros	72 (37.3)	74.5	
>3600 euros	6 (3.1)	61.3	
*Source of Income*			
Salaried	130 (69.5)	70.78	F 4.977 *p* < 0.001
Self-employed	19 (10.2)	67.3	
Help from relatives	8 (4.3)	84	
Subsidies	30 (16)	63.8	
*Pregnancies*			
No	93 (46.5)		F 4.137 *p* < 0.004
1	43 (21.5)	68.1	
2	49 (24.5)	69.7	
>3	15 (7.5)	66.2	
*Use of ART*			
Yes	47 (21.4)	72.6	
No	173 (78.6)	70.46	
*Current Depression*			
Yes	47 (21.4)	64.02	F 4.146 *p* < 0.045
No	137 (62.3)	69.75	
*Attending Endometriosis Associations*			
Yes	77 (39.6)	56.6	F 8.3 *p* < 0.004
No	117 (59.6)	70.59	

**Table 4:** Mean scores of psychometric scales according to the level of resilience using CDRISC-25. Low resilience: scores below the first quartile (Q1: 59.75). High resilience: scores higher than the third quartile (Q3: 80.25).

CD-RISC25		STAI (State)	STAI (Trait)	BDS	SF-36 Physical	SF-36 Mental	FSFI
*Low Resilience*	Mean	24.8	27.4	5.8	44.2	43.9	3.8
	Std. Dev	5.8	4.5	4.3	9.5	10.1	1.2
*High Resilience*	Mean	26.1	24.1	2.6	45.1	49.5	3.5
	Std. Dev	6.7	7.5	2.5	8.0	10.4	0.8
*p-Value*		0.02	0.00	0.00	ns	0.01	ns
*Total*	Mean	24.5	24.5	3.8	44.8	47.5	3.5
	Std. Dev	5.6	6.1	3.2	9.6	9.8	1.0

**Table 5:** Mean scores of the visual-analog pain scales as a function of the resilience level obtained with CDR-25. Low resilience: scores below the first quartile (Q1: 59.75 in CDR-25). High resilience: scores higher than the third quartile (Q3: 80.25 in CDR-25).

Visual-Analog Pain Scales (0–10)	CDR-25		
	LR	HR	*p*-Value
Dysmenorrhea	7.58 [2.54]	7.52 [2.34]	NS
	*Cohen’s D* = 0.14		
Abdominal Pain	7.03 [1.86]	6.19 [2.24]	F4.5 *p* < 0.05
	*Cohen’s D* = 0.71		
Dyspareunia	6.57 [2.36]	5.23 [2.53]	F: 6.27 *p* < 0.014
	*Cohen’s D* = 0.67		
Dyschezia	5.70 [3.09]	5.49 [2.52]	NS
	*Cohen’s D* = 0.12		
Dysuria	4.94 [2.38]	5.0 [2.59]	NS
	*Cohen’s D* = −0.08		

**Table 6:** Predictive model for resilience. Goodness of fit indexes.

Fit Statistics	Values	90% Confidence Interval
Likelihood ratio chi2_ms	0.178 model vs. saturated	
*p* > chi2	0.673	
Root mean squared error of approximation (RMSEA)	0.001	0.000–0.230
Akaike’s information criterion (AIC)	1431.977	
Bayesian information criterion BIC	1443.564	
Comparative fit index (CFI)	1.000	
Tucker–Lewis index (TLI)	1.322	
Standard root mean squared residual (SRMR)	0.012	
Coefficient of determination (CD)	0.190	

## Supplementary Materials

The following are available online at https://www.mdpi.com/article/10.3390/jcm10245942/s1, Figure S1, Structural model for resilience; Table S1, Goodness of fit statistics for the five-factors model.
