# Supplementary material for: Measuring Resilience in Women with Endometriosis
Source: J Clin Med. 2021 Dec 17;10(24):5942. doi: 10.3390/jcm10245942 (PMC8708759; doi:10.3390/jcm10245942)
Supplement: Supplementary file 1 [file jcm-10-05942-s001.zip › jcm-1492029-supplementary.pdf]

Supplementary materials: Validation of the Resilience Scales

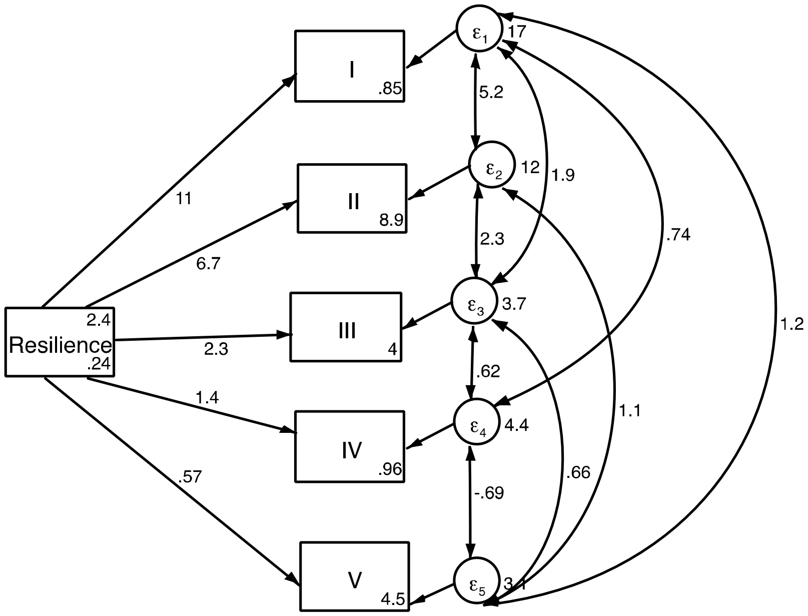

Figure S1. Structural model for resilience.

Table S1. Goodness of fit statistics for the five-factors model.

Fit Statistics	Values	90% Confidence Interval
Likelihood ratio chi2_ms	1.710 model vs. saturated	
p > chi2	0.191	
Root mean squared error of approximation (RMSEA)	0.065	0.000–0.22
Akaike's information criterion (AIC)	4178.895	
Bayesian information criterion (BIC)	4254.013	
Comparative fit index (CFI)	10.998	
Tucker–Lewis index (TLI)	0.973	
Standard root mean squared residual (SRMR)	0.016	
Coefficient of determination (CD)	0.667	
